# Trafficking modulator TENin1 inhibits endocytosis, causes endomembrane protein accumulation at the pre-vacuolar compartment and impairs gravitropic response in *Arabidopsis thaliana*

**DOI:** 10.1042/BJ20131136

**Published:** 2014-05-13

**Authors:** Rupesh Paudyal, Adam Jamaluddin, James P. Warren, Siamsa M. Doyle, Stéphanie Robert, Stuart L. Warriner, Alison Baker

**Affiliations:** *Centre for Plant Sciences, University of Leeds, Leeds LS2 9JT, U.K.; †School of Chemistry, Faculty of Mathematics and Physical Sciences, University of Leeds, Leeds LS2 9JT, U.K.; ‡Umeå Plant Science Centre, Department of Forest Genetics and Plant Physiology, Swedish University of Agricultural Sciences (SLU), Umeå 90183, Sweden

**Keywords:** chemical biology, endocytosis, gravitropism, PIN-FORMED protein (PIN protein), trafficking and endocytosis inhibitor 1/TENin1 (TE1), *trans*-Golgi network (TGN), ABD2, actin-binding domain 2, ARA7, *Arabidopsis* Rab GTPase homologue F2B, BFA, Brefeldin A, BR, brassinosteroid, BRI1, BR (receptor) insensitive 1, ES1, endosidin1, GEF, GTP-exchange factor, LatB, latrunculin B, LPVC, late PVC, MS medium, Murashige and Skoog medium, NAA, 1-naphthaleneacetic acid, NAG, *N*-acetylglucosaminyltransferase, PI3K, phosphoinositide 3-kinase, PIN, PIN-FORMED, PIP2a, PM intrinsic protein 2a, PM, plasma membrane, PVC, pre-vacuolar compartment, Rha1, *Arabidopsis* Rab homologue F2A, SAR, structure–activity relationship, secGFP, secreted GFP, TE1, trafficking and endocytosis inhibitor 1/TENin1, TGN, *trans*-Golgi network, VHAa1, vacuolar H^+^-ATPase subunit a1

## Abstract

Auxin gradients are established and maintained by polarized distribution of auxin transporters that undergo constitutive endocytic recycling from the PM (plasma membrane) and are essential for the gravitropic response in plants. The present study characterizes an inhibitor of endomembrane protein trafficking, TE1 (trafficking and endocytosis inhibitor 1/TENin1) that reduces gravitropic root bending in *Arabidopsis thaliana* seedlings. Short-term TE1 treatment causes accumulation of PM proteins, including the BR (brassinosteroid) receptor BRI1 (BR insensitive 1), PIP2a (PM intrinsic protein 2a) and the auxin transporter PIN2 (PIN-FORMED 2) in a PVC (pre-vacuolar related compartment), which is sensitive to BFA (Brefeldin A). This compound inhibits endocytosis from the PM and promotes trafficking to the vacuole, consistent with inhibition of retrieval of proteins to the TGN (*trans*-Golgi network) from the PVC and the PM. However, trafficking of newly synthesized proteins to the PM is unaffected. The short-term protein trafficking inhibition and long-term effect on plant growth and survival caused by TE1 were fully reversible upon drug washout. Structure–activity relationship studies revealed that only minor modifications were possible without loss of biological activity. Diversity in *Arabidopsis* ecotypes was also exploited to identify two *Arabidopsis* accessions that display reduced sensitivity to TE1. This compound and the resistant *Arabidopsis* accessions may be used as a resource in future studies to better understand endomembrane trafficking in plants.

## Abstract 2

In the present study a detailed characterization of a small molecule inhibitor of protein trafficking and gravitropic response is described. We also identified two *Arabidopsis thaliana* ecotypes that display resistance to this compound. The ecotypes and chemical provide useful tool to investigate protein trafficking.

## INTRODUCTION

Auxin regulates diverse developmental and growth processes as well as tropic responses [1]. As the Cholodny–Went theory proposes, polarized auxin distribution has been shown to be one of the major factors that regulate the gravitropic response in plants [2]. Gradients formed by polar auxin transport are predominantly regulated by PIN (PIN-FORMED) proteins for auxin efflux. Different members from the family of PIN proteins operate in different cell types, and show distinct polarization in the PM (plasma membrane) according to the cell type. In the *Arabidopsis thaliana* root, PIN1 and PIN2 are polarized basally (‘rootward’) in the stele and young cortex respectively, PIN2 is apical (‘shootward’) in the epidermis, and PIN3 and PIN7 are apolar in the columella [3].

Polarized distribution of PINs is regulated by constitutive endocytic recycling to and from the PM [4] via clathrin-mediated endocytosis [5] and ARF (ADP-ribosylation factor)-GEF (GTP-exchange factor)-dependent exocytosis. The activity of the fungal toxin BFA (Brefeldin A) inhibits endocytic recycling, inducing the formation of intracellular agglomerations known as BFA bodies [6,7]. Endosome-resident membrane proteins can also be sorted to the PVC (pre-vacuolar compartment) and targeted to the lytic vacuole for degradation [8]. This process is dependent on PI3K (phosphoinositide 3-kinase), whose function is blocked by wortmannin causing interference with membrane trafficking [9].

Dissecting rapid processes such as signalling and trafficking mechanisms is challenging for classical genetics experiments. After mutagenesis, the resulting phenotype reports the status of the cells in equilibrium with a lesion. Moreover, conventional genetics cannot address problems of gene redundancy and lethality. Therefore the use of small molecules to modify or disrupt the function of a specific protein can help us to fully understand the complexity of endomembrane trafficking in plants. The power of this approach is the ability to study protein function with precise control of response via bioactive chemicals [10]. A high-throughput chemical screen identified ES1 (endosidin1), which displays selective inhibition of membrane transporter trafficking, causing them to agglomerate at the TGN (*trans*-Golgi network) [11]. This demonstrates the use of chemical genomics as a powerful tool to isolate small molecules to unpick different reactions and pathways in a model system.

The present study characterizes a new small molecule TE1 (trafficking and endocytosis inhibitor 1/TENin1) that inhibits endocytosis from the PM and interferes with protein retrieval from the PVC to the TGN and causes agravitropic growth in *Arabidopsis* seedlings.

## EXPERIMENTAL

### Plant materials, growth and experimental conditions

*Arabidopsis* seeds sterilized and stratified at 4°C for 48 h were sown on 0.5× MS medium (Murashige and Skoog medium) containing 0.8% plant agar and grown in 16 h light or in total darkness at 22°C. The *Arabidopsis* lines used expressed the following proteins: PIN2–GFP from [12], GFP–ARA7 (*Arabidopsis* Rab GTPase homologue F2B) from [13], GFP–ABD2 (actin-binding domain 2) from [14], NAG (*N*-acetylglucosaminyltransferase)–GFP from [15], VHAa1 (vacuolar H^+^-ATPase subunit a1)–GFP from [16], BRI1 [BR (brassinosteroid) (receptor) insensitive 1]–GFP from [17], PIP2a (PM intrinsic protein 2a)–GFP from [18] and secGFP (secreted GFP) from [19].

For growth and germination/survival assays seedlings were grown for 7 days. For gravitropism, 6-day-old seedlings were transplanted to medium containing TE1 and rotated 90° for 48 h. Root angle after gravistimulation and root/hypocotyl length were measured using ImageJ (http://imagej.nih.gov/ij/). For the natural accession screen, an *Arabidopsis* accession from the 1001 genome project, N76427, was purchased from Nottingham *Arabidopsis* Stock Centre.

### Chemical treatments

Seedlings (7–8-day-old) were treated on MS medium containing following concentrations (unless specified otherwise): 25 μM TE1 (Sigma–Aldrich) for 120 min, 50 μM BFA for 60 min, 5 μM NAA (1-naphthaleneacetic acid) and 100 nM LatB (latrunculin B). For gravitropic screening using natural accessions, LatB was used at 10 nM and 50 nM. To trace endocytosis, seedlings were incubated in 5 μM ice-cold FM4-64 in MS medium for 5 min and promptly visualized by confocal microscopy.

### Imaging and immunolabelling

Immunolabelling using an anti-PIN2 antibody [12] was performed as described in [20]. For details of the immunolabelling and confocal imaging see the Supplementary Online Data (http://www.biochemj.org/bj/460/bj4600177add.htm)

## RESULTS

### TE1 inhibits plant growth and gravitropism

The compound TE1 was identified as an inhibitor of plant hypocotyl gravitropism by a large-scale chemical genomic screen [21]. Hypocotyl lengths in dark-grown *Arabidopsis* seedlings were reduced by 30% in the presence of 1 μM TE1. Dose-dependent reduction in hypocotyl length was observed at 5 μM and 10 μM, but higher concentrations did not further reduce the hypocotyl length (Supplementary Figure S1A at http://www.biochemj.org/bj/460/bj4600177add.htm). Root growth in the light-grown seedlings was less sensitive, with no effect observed at 1 μM TE1; however, 75% root growth inhibition was observed at 50 μM TE1 (Supplementary Figure S1A). Germination was unaffected by TE1, but at concentrations above 10 μM necrotic seedlings were observed after 7 days (Supplementary Figures S1B and S8A, top row).

An advantage of chemical biology methods is the possibility to test the reversibility of the chemical activity in a given system. Seedlings were severely retarded in growth after 5 days in 25 μM TE1 (Supplementary Figure S1D) compared with the controls (DMSO-treated; Supplementary Figure S1C), but the effects could be reversed by transplanting to a compound-free medium (Supplementary Figure S1F). Control plants transplanted into a fresh DMSO-containing medium for a further 5 days continued normal growth (Supplementary Figure S1G), whereas almost all of the plants transplanted from TE1 to medium containing TE1 for a further 5 days were dead (Supplementary Figure S1E). Overall, these results indicate that TE1 affects the post-embryonic developmental mechanisms regulating seedling growth in a dose-dependent and reversible manner.

**Figure 1:**
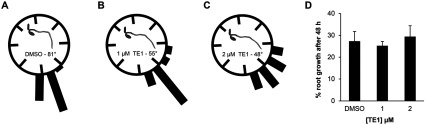
TE1 decreases the root gravitropic response Seedlings (6-day-old) were transplanted into medium containing DMSO (**A**), 1 μM TE1 (**B**) or 2 μM TE1 (**C**) for 48 h and gravistimulated at 90°. The distribution and root bending in response to gravistimulation after 48 h is indicated. Root growth after transplantation into DMSO, 1 μM TE1 or 2 μM TE1 for 48 h is also shown (**D**). The results represent a typical dataset from three repetitions, *n*=15–24 seedlings per condition. Error bars represent the S.E.M.

TE1 inhibited the hypocotyl gravitropic response in a large chemical screen [21]; however, the present study shows it compromises both hypocotyl and root growth. Therefore gravitropism was tested in roots at TE1 concentrations which did not affect root growth. Seedlings (6-day-old) were transplanted into medium containing DMSO, 1 μM TE1 or 2 μM TE1 and gravistimulated at 90° for 48 h. Untreated roots showed an average bending of 81°, whereas only 55° and 48° bending was observed in seedlings treated with 1 μM and 2 μM TE1 respectively (Figures 1A–1C, and Supplementary Figure S2 at http://www.biochemj.org/bj/460/bj4600177add.htm). Root growth in 1 μM and 2 μM TE1 after 48 h was similar to that of the DMSO control (Figure 1D) and, therefore, the impaired gravitropic response was not due to the inhibition of root growth which requires a 5 μM or higher concentration of TE1 (Supplementary Figure S1A).

### TE1 inhibits endomembrane protein trafficking

Gravitropic response has previously been shown to be mediated by endomembrane trafficking [1]. In particular, PIN2 proteins have been shown to play an important part in the regulation of gravitropism in *Arabidopsis* [22–25]. Therefore the hypothesis that TE1 may affect intracellular distribution of PIN2 in *Arabidopsis* roots was tested.

**Figure 2:**
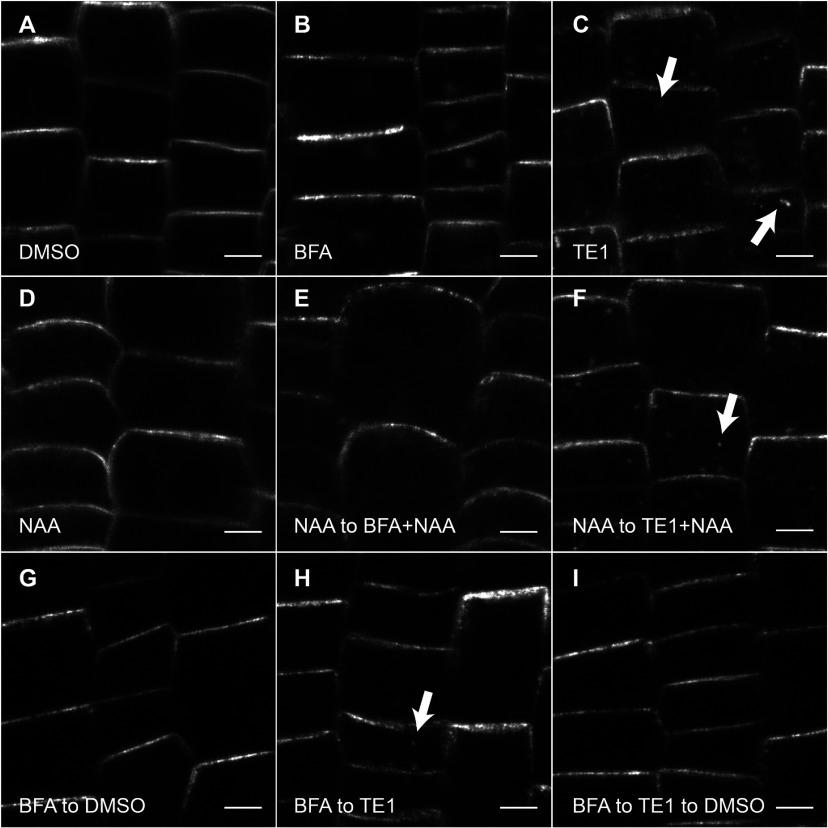
TE1 interferes with membrane protein trafficking Seedlings expressing PIN2–GFP were transplanted into DMSO control (**A**), 50 μM BFA for 60 min (**B**), 25 μM TE1 for 120 min (**C**), 5 μM NAA for 150 min (**D**), 50 μM BFA and 5 μM NAA for 60 min following pre-treatment for 30 min with 5 μM NAA (**E**), 25 μM TE1 and 5 μM NAA for 120 min following a pre-treatment with 5 μM NAA for 30 min (**F**), DMSO for 120 min after 60 min with 50 μM BFA (**G**), 25 μM TE1 for 120 min after 60 min with 50 μM BFA (**H**), or DMSO for 180 min following 60 min with 50 μM BFA and 120 min with 25 μM TE1 (**I**). Scale bars, 5 μm.

In the root epidermis of untreated seedlings, PIN2 proteins are localized to the apical PM (Figure 2A). Seedlings treated with 25 μM TE1 for 120 min caused accumulation of PIN2–GFP in intracellular bodies (in the present paper termed TE1 bodies) (Figure 2C, arrows). Similarly, TE1 also increased the intracellular accumulation of other recycling proteins that display non-polarization at the PM, such as the BR receptor BRI1 and PIP2a (Supplementary Figure S3 at http://www.biochemj.org/bj/460/bj4600177add.htm). These results show that TE1 interferes with trafficking of all these membrane proteins, regardless of whether they localize apically or basally or if they are non-polarized in the PM. Therefore PIN2–GFP was used as a model for further investigation.

PIN2 localization is determined by several trafficking mechanisms including endocytosis from the PM [26]. Auxin has been shown to inhibit clathrin-mediated endocytosis of PIN proteins [26,27]. To assess the role of TE1 in endocytic membrane recycling, BFA and auxin were used as pharmacological tools to modulate the specific stages of endocytic protein recycling (Figure 2). BFA caused accumulation of PIN2 in BFA bodies (Figure 2B) and this could be prevented by pre-treating the seedlings with NAA (Figure 2E) [26]. In contrast, inhibition of PIN2 internalization with NAA did not prevent accumulation of PIN2 in TE1 bodies (Figure 2F), indicating that PIN2 proteins agglomerating in TE1 bodies did not originate from the PM. Average PIN2–GFP intensity at the PM was also quantified and normalized against DMSO (*n*=87). Average PIN2–GFP intensity at the PM in samples treated with TE1 (*n*=115) was 101±3% relative to the control seedlings and the differences were not statistically significant.

To determine whether the TE1-induced intracellular accumulations were observed due to the inhibition of membrane protein trafficking to the PM, the effect of TE1 on exocytosis was monitored by recovery of BFA bodies in the presence of TE1. PIN2-labelled BFA bodies were no longer visible in seedlings transplanted from BFA to DMSO for 120 min (Figure 2G). Similarly, BFA bodies, marked by PIN2–GFP, were recovered to TE1 bodies in seedlings transplanted to TE1 following BFA treatment (Figure 2H). Intracellular PIN2 accumulation could also be reversed in seedlings transplanted to DMSO for 180 min after BFA and TE1 treatments respectively (Figure 2I), indicating that the TE1 effect was reversible. As PIN2–GFP is recovered from BFA bodies in the presence of TE1, this argues against the inhibition of TGN to PM trafficking. To reinforce this, secretion of newly synthesized protein was also investigated using secGFP lines. As expected, a weak GFP signal was observed in the apoplast under control conditions (Supplementary Figure S4A at http://www.biochemj.org/bj/460/bj4600177add.htm) [19]. Similarly, TE1 did not interfere with the trafficking of secGFP to the apoplast (Supplementary Figure S4C); however, BFA used as a control caused intracellular accumulation of secGFP (Supplementary Figure S4B).

To gain insight into the structural features of TE1 that are important for activity, a SAR (structure–activity relationship) study was performed. In a small-scale screen, analogues of TE1 were synthesized in the laboratory and PIN2–GFP seedlings were incubated in 25 μM chemical for 120 min to monitor the change in PIN2 localization. As shown in Supplementary Figure S5 (http://www.biochemj.org/bj/460/bj4600177add.htm), the SAR studies indicated that JW4, JW42, JW45, JW47 and JW48 are inactive. However, JW22, JW30, JW32 and JW35 displayed similar effect on PIN2–GFP distribution to that caused by TE1. This shows that any major modification to the compound causes a loss in biological activity.

### TE1 inhibits FM4-64 uptake and increases vacuolar labelling

**Figure 3:**
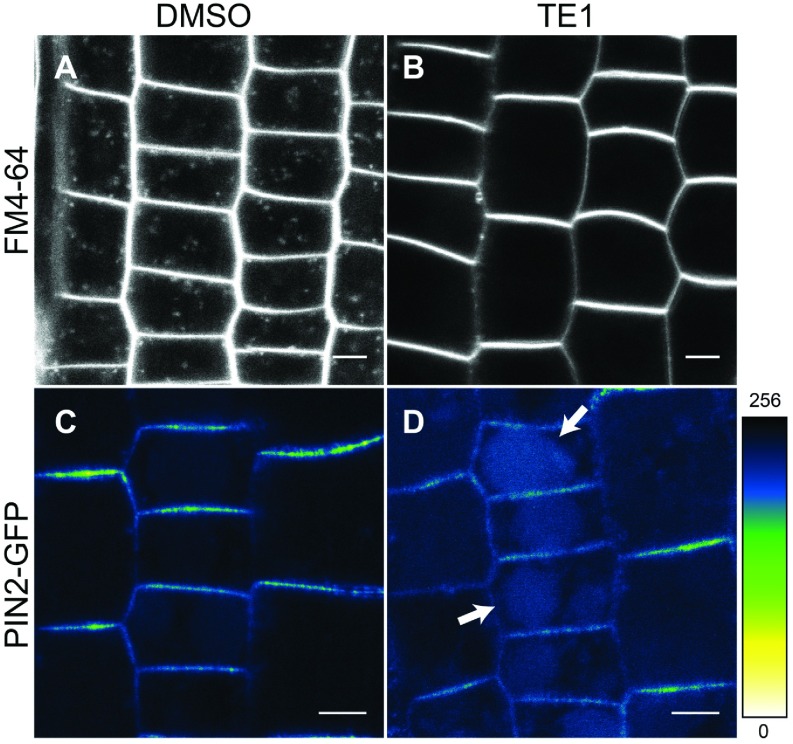
TE1 inhibits endocytosis and increases vacuolar labelling Wild-type Col-0 seedlings were stained with FM4-64 and visualized after 15 min following a 120 min incubation in DMSO (**A**) and 25 μM TE1 (**B**). (**C** and **D**) Heat map of PIN2–GFP in seedlings incubated in DMSO (**C**) and 25 μM TE1 (**D**) for 6 h in the dark. The colour panel on the bottom right-hand side displays the relative intensity of PIN2–GFP (**C** and **D**) from 0–256. Scale bars, 5 μm.

As discussed above, Figure 2 suggests that PIN2 proteins within the TE1 bodies do not originate from the PM. Therefore the effect of TE1 on endocytosis was monitored through the uptake of the endocytic tracer FM4-64, which binds to membrane lipids [28]. Seedlings treated with 25 μM TE1 for 120 min prevented the uptake of FM4-64 (Figures 3A and 3B). This result suggests that TE1 affects the early steps of the endocytosis pathway from the PM.

The effect of TE1 on vacuolar trafficking was also tested by taking advantage of GFP stability in the lytic vacuole in the dark [29]. Some lytic vacuole labelling with PIN2–GFP was observed in the DMSO control at 6 h in the dark (Figure 3C). However, in seedlings treated with 25 μM TE1 for 6 h in the dark, the intensity of GFP in the lytic vacuole was higher compared with the control (Figures 3C and 3D, arrows). Increased vacuolar accumulation of PIN2 after prolonged exposure to TE1 suggests that protein retrieval from the late vacuolar trafficking step is also affected by TE1.

### Immunolocalization shows that TE1 bodies are derived from the PVC

**Figure 4:**
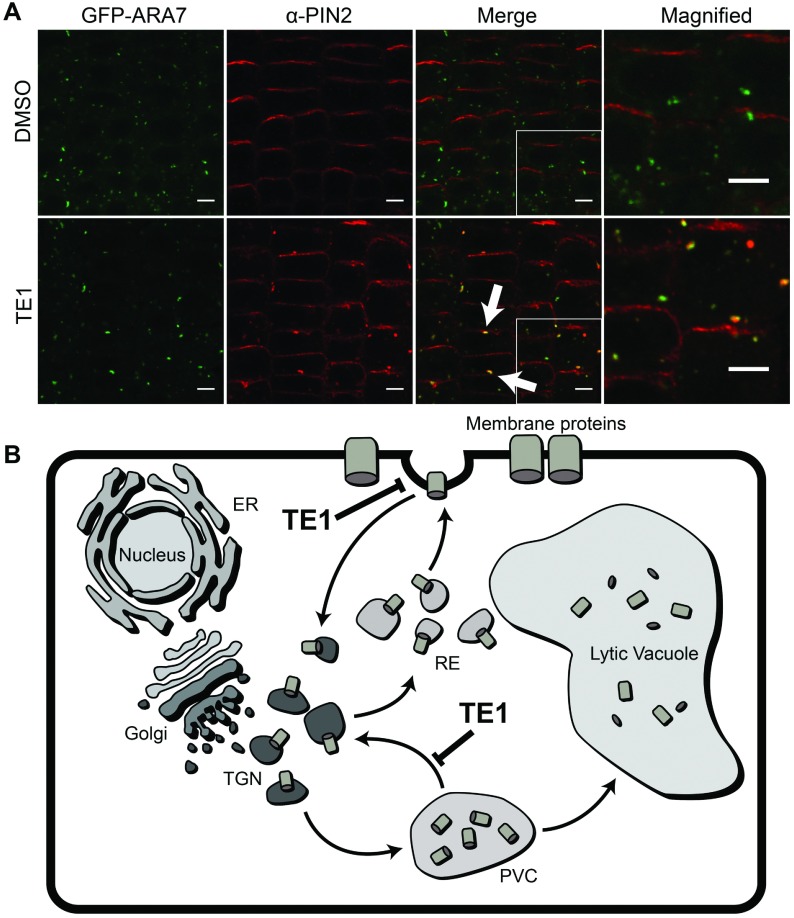
TE1 causes protein accumulation at the PVC Seedlings expressing GFP–ARA7 were incubated in DMSO (upper row) and 25 μM TE1 (lower row) for 120 min. The first column shows GFP–ARA7 and the second column shows immunolabelling of PIN2. The last two columns are merged images of GFP–ARA7 (the last column is a magnification of a section of the third column) and labelling with anti-PIN2 antibody (**A**). Scale bars, 5 μm. (**B**) Endomembrane protein trafficking and possible mode of action of TE1.

To identify the endomembrane compartment in which PIN2–GFP accumulates in the presence of TE1, immunolocalization using an anti-PIN2 antibody was performed in lines expressing NAG–GFP (targeted to Golgi), GFP–ARA7 (for the PVC) and VHAa1–GFP (for the TGN population). Immunolabelling revealed that native PIN2 protein behaves in the same way as the PIN2–GFP in stably transformed *Arabidopsis* lines (Figure 4A). TE1 bodies identified by anti-PIN2 did not co-localize with NAG–GFP or VHAa1–GFP (Supplementary Figure S6 at http://www.biochemj.org/bj/460/bj4600177add.htm); however, TE1 bodies co-localized with the GFP–ARA7 compartments (Figure 4A, arrows), indicating that TE1 bodies contain PVC. PIN2–GFP seedlings were also pre-incubated in TE1 (showing TE1 bodies) followed by TE1 and BFA treatment (Supplementary Figure S7 at http://www.biochemj.org/bj/460/bj4600177add.htm). The TE1 bodies in these seedlings were replaced with structures similar in appearance to the BFA bodies. This result indicates that the TE1 bodies are sensitive to the effects of BFA. It is been demonstrated previously that the PVCs labelled by ARA7 are sensitive to the effects of BFA [30]. Accumulation of proteins at the PVC suggests that vacuolar trafficking pathway is being affected by TE1.

Overall the results suggest that TE1 interferes with trafficking to the TGN, either from the PM or the PVC, therefore protein accumulation is visible in the PVC after short-term TE1 treatment. However, a prolonged exposure to TE1 leads protein accumulation to the vacuole. Taken together the findings lead to the proposed model shown in Figure 4(B).

### Effects of TE1 on the actin cytoskeleton

Intracellular agglomeration of membrane proteins has been widely reported upon depolymerization of the actin cytoskeleton, therefore the effects of TE1 on actin were investigated. In the presence of 25 μM TE1 for 120 min actin filaments, visualized by GFP–ABD2, were comparable with the DMSO control showing fine filaments of the actin network throughout the cell (Supplementary Figures S8A and S8B at http://www.biochemj.org/bj/460/bj4600177add.htm). In contrast, 100 nM LatB depolymerized the actin network (Supplementary Figure S8C). Intracellular PIN2–GFP accumulation was still observed when seedlings were treated with 100 nM LatB (Supplementary Figure S8F).

**Figure 5:**
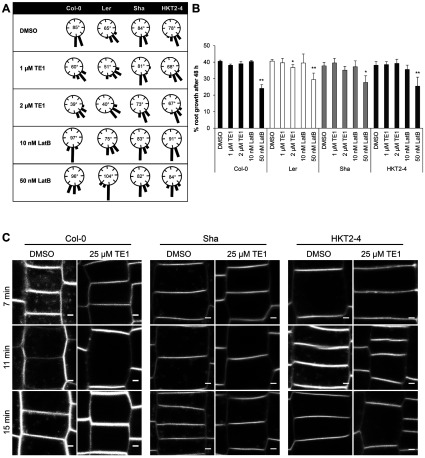
*A. thaliana* natural accession screen reveals two ecotypes that display reduced sensitivity to TE1 Col-0, L*er*, Sha and HKT2-4 seedlings (6-day-old) grown in DMSO control were transplanted and gravistimulated at 90° for 48 h in presence of DMSO, 1 μM TE1, 2 μM TE1, 10 nM LatB or 50 nM LatB. (**A**) Histograms of root bending. The number in the middle of the histograms shows the average angle root bending after 48 h of gravistimulation. (**B**) Percentage root growth after 48 h of transplant. Error bars represent the S.E.M. *P≤0.05 and **P≤ 0.01. (**C**) Col-0, Sha and HKT2-4 seedlings were incubated in DMSO or 25 μM TE1 for 120 min, and imaged 7, 11 and 15 min after FM4-64 staining. Scale bars, 2 μm.

Decreased gravitropic bending after 48 h was observed in concentrations of TE1 as low as 1 μM (Figures 1 and 5A); however, no visible change in actin was observed after 48 h of treatment of TE1 up to a 10 μM concentration (Supplementary Figure S8E). Depolymerization of the actin cytoskeleton by LatB slightly increased the root bending up on gravistimulation (Figure 5A). Severe reduction of growth was observed in the presence of 50 nM LatB, but root growth was comparable with that of the DMSO control in 10 nM LatB (Figure 5B). These data indicate that a 120-min treatment with 25 μM TE1 does not cause depolymerization of the actin filaments.

### Identification of two *Arabidopsis* ecotypes that display reduced sensitivity to TE1

*A. thaliana* is an excellent model to study natural variation because of the sequenced genome and extensive resources [31]. Genome information is now available for 80 ecotypes [32]. These 80 ecotypes, along with Columbia (Col-0), Landsberg erecta (L*er*) and Wassilewskija (Ws2), were screened to identify ecotypes that display an altered sensitivity to TE1 (Supplementary Table S1 at http://www.biochemj.org/bj/460/bj4600177add.htm). Primary screening selected for ecotypes that displayed a germination rate higher than 50%, with an average root length of 20±5 mm in 7-day-old seedlings. A secondary screen identified ecotypes that displayed a reduced sensitivity to agravitropic growth caused by 2 μM TE1. Root growth of all 39 ecotypes in the secondary screen at 48 h after transplant in to 2 μM TE1 was comparable with that of the DMSO control. The secondary screen yielded 20 ecotypes that displayed some resistance to agravitropic growth caused by TE1. These 20 ecotypes were re-tested in a tertiary screen that confirmed that Shahdara (Sha) and Heiligkreuztal 2 (HKT2-4) have a reduced sensitivity to the effects of TE1 (Supplementary Table S1).

After transplant and gravistimulation at 90° for 48 h, the sensitive *Arabidopsis* ecotypes Col-0 and L*er* displayed 70% (60°/85°) and 78% (51°/65°) root bending in 1 μM TE1 compared with their controls. However, the resistant ecotypes Sha and HKT2-4 showed 96% (81°/84°) and 87% (68°/78°) gravity response respectively relative to the control. In 2 μM TE1, Sha and HKT2-4 showed 86% and 85% of the control response, but Col-0 and L*er* showed 45% and 61% (Figure 5A). In the presence of LatB, no inhibition of gravitropism was seen in any line at either 10 or 50 nM (Figure 5A), although LatB inhibited root growth in all accessions at 50 nM. Sha and HKT2-4 also showed much reduced growth inhibition after growth for 7 days in 10 μM TE1 and 25 μM TE1, although growth at these higher concentrations was agravitropic (Supplementary Figure S9 at http://www.biochemj.org/bj/460/bj4600177add.htm). Sha and HKT2-4 were also less sensitive to the inhibition of FM4-64 uptake by TE1. In these partially resistant ecotypes some intracellular FM4-64 labelling was observed after 11 and 15 min (Figure 5C and Supplementary Figure S9C). Sha and HKT2-4 seedlings were also monitored for TE1-induced intracellular agglomerations detected by anti-PIN2 antibodies (Supplementary Figure S10 at http://www.biochemj.org/bj/460/bj4600177add.htm). TE1 bodies were detected in 28 out of 36 Col-0 seedlings (pooled data from three independent experiments), whereas TE1 bodies were only detected in 11 out of 37 Sha seedlings and 10 out of 31 HKT2-4 seedlings from three independent experiments. Therefore Sha and HKT2-4 seedlings also showed increased resistance to the effects of TE1 in inducing intracellular agglomerations, relative to Col-0, as detected by anti-PIN2 antibodies (Supplementary Figure S10).

Collectively, these results show that TE1 does not have obvious effects on the actin cytoskeleton at the concentrations used, and actin depolymerization does not inhibit gravitropism. Sha and HKT2-4 show reduced phenotypes caused by TE1, but similar phenotypes are still seen upon actin depolymerization. Therefore TE1 has a distinct mode of action to LatB and these accessions are useful tools to investigate the effects of this chemical in the future.

## DISCUSSION

The present study characterized the cellular and whole-plant effects of TE1. It demonstrates that short-term response to exposure to TE1 results in the inhibition of endocytosis (Figure 3B and Supplementary Figure S9C) and accumulation of PIN2–GFP in a pre-vacuolar-derived compartment (Figure 4A). The results of the present study led to the model shown in Figure 4(B) and discussed in detail below. This cellular phenotype would explain the reduced gravitropic response caused by TE1 (Figures 1 and 5A). The decreased gravitropic response is not due to growth inhibition as exposure of seedlings to 1 μM and 2 μM TE1 for 48 h or even 7 days had no effect on root length, but still reduced root bending (Figure 1 and Supplementary Figure S2). TE1 is not a specific inhibitor of PIN2 trafficking since other membrane proteins, such as BRI1 and PIP2a, were also affected (Supplementary Figure S3). This general effect on membrane protein trafficking could explain the severe, eventually lethal, effects of chronic long-term TE1 exposure (Supplementary Figure S1).

TE1 inhibits root growth in light and hypocotyl elongation in the dark (Supplementary Figure S1); however, TE1 was also identified in a screen for compounds that increase hypocotyl length in plants grown in light [33]. This apparent contradiction can be explained by the finding that that auxin transport is required for hypocotyl growth in light grown seedlings, but not in etiolated seedlings [34].

SARs of TE1 were examined by testing the effects of a focused library of TE1 derivatives on PIN2–GFP trafficking (Supplementary Figure S5). Taken together, the SAR studies show that the carbazole core structure is crucial for bioactivity of TE1, and both the triazole head group and the alkyl chain make contributions although the latter two regions can accommodate minor modifications.

Small molecule inhibitors of endomembrane trafficking are widely reported to affect gravitropism and plant growth; however, the cellular effects of TE1 can be distinguished from other compounds such as BFA [4], ES1 [11] and wortmannin [9]. BFA treatment resulted in intracellular accumulation of PIN2–GFP in structures that were distinct in appearance from those formed upon treatment with TE1. Intracellular accumulation of PIN2–GFP caused by BFA could be prevented by pre-treatment with auxin as reported previously [26]. However, in TE1-treated cells PIN2–GFP still accumulated intracellularly in the presence of auxin (Figure 2F), whereas uptake of endocytic tracer FM4-64 was inhibited (Figure 3B). These results suggest that proteins recycling from the PM do not significantly contribute to the formation of TE1 bodies. BFA bodies could also be rescued upon transplantation to TE1 to be replaced by TE1 bodies (Figure 2H). This result suggests that TE1 does not affect TGN-to-PM exocytosis, also supported by the finding that the secretion of secGFP is unaffected by TE1 (Supplementary Figure S4).

PIN2 trafficking to the vacuole is inhibited with wortmannin treatment [8]; however, higher GFP content in the lytic vacuole is observed in PIN2–GFP seedlings treated with TE1 in the dark compared with the non-treated seedlings. This result suggests that PI3K, required for vacuolar trafficking, is fully functional in the presence of TE1, which has a different mode of action to that of wortmannin. Trafficking to the vacuole may be up-regulated after prolonged exposure to TE1 (Figures 3C and 3D), which could disrupt the differential degradation of PIN2 protein that is required for gravitropic response [8,12]. However, it is most probable that if the retrieval of proteins to the TGN compartments from the PVC is inhibited, then both newly synthesized proteins and those cycling between intracellular endomembrane compartments may be redirected towards the vacuole (Figure 3D). This is supported by the finding that TE1-induced intracellular PIN2 accumulation co-localizes with GFP–ARA7, a known PVC marker (Figure 4). TE1 bodies are also replaced by BFA-like bodies upon transplant of seedlings from TE1 to medium containing TE1 and BFA (Supplementary Figure S7), which suggests that TE1 bodies are sensitive to BFA. This is consistent with previous findings that show that compartments labelled by ARA7 are sensitive to the effects of BFA [30].

Taken together, the results of the present study suggest that the protein observed in intracellular compartments in the presence of TE1 has been accumulated from proteins already within the endomembrane system before the addition of TE1. This is consistent with TE1 inhibiting two distinct steps of membrane trafficking, endocytosis and recycling from the PVC to the TGN resulting in the increased direction of PIN2–GFP to the vacuole over a period of several hours (Figure 3D).

ARA7 belongs to the Rab5 family of Rab GTPases and all of the Rab5 members in *Arabidopsis* may be activated by the GEF VPS9a (vacuolar protein sorting-associated protein 9A) [35]. A GDP-locked version of ARA7 also had an inhibitory effect on the endocytic uptake of FM4-64 [36]. Previously a matured form of PVC, termed the LPVC (late PVC), was shown to act as an intermediate compartment between the PVC and the vacuole in tobacco leaf epidermis [37]. However, LPVC has not been reported in *Arabidopsis* to date. Co-localization of the Rab5 homologues, Rha1 (*Arabidopsis* Rab homologue F2A) and ARA7, revealed they both localize to the PVC [38], and recently Rha1 was shown to be localized to the LPVC in tobacco [39]. Overexpression of Rab5 GTPases was also reported to cause fusion of TGN with the PVC [39]; similarly, wortmannin treatment also caused fusion of PVCs [40]. Although we cannot exclude the possibility that TE1 also affects the distribution of ARA7, the appearance of this compartment was not obviously different compared with the control (Figure 4A). Moreover, in the presence of TE1, no homotypic fusion of TGN with the PVC subpopulation was observed, as TE1-induced compartments did not co-localize with NAG–GFP or VHAa1–GFP (Supplementary Figure S6). However, TE1 bodies when in the presence of BFA were replaced with structures resembling BFA bodies (Supplementary Figure S7).

The compound ES1 interferes with the trafficking of BRI1, and apically localized AUX1 and PIN2, whereas basally localized PIN1 and non-polar PIN7 and PIP2a are unaffected [11]. The compound TE1, however, affects the trafficking of PIN2, BRI1 and PIP2a. Interference of ES1 with endomembrane trafficking has recently been linked to the stabilization of actin filaments caused by the compound [41]. However, stabilization of the actin cytoskeleton by ES1 only selectively disrupts endomembrane trafficking, and does not alter the gravitropic response of plants [11].

In contrast, TE1 does not have a striking visual effect on the organization of the actin cytoskeleton either on short-term exposure to 25 μM TE1 or 48 h exposure to 10 μM TE1 (Supplementary Figure S9). Yet the former results in accumulation of PIN2–GFP in the PVC (Figure 4) and the latter is an order of magnitude higher than the concentration required to affect gravitropism (Figures 1 and 5). Depolymerization of the actin cytoskeleton was observed in the presence of 100 nM LatB (Supplementary Figure S8). However, it was also observed that even slight depolymerization of actin filaments increases root bending in response to gravistimulation (Figure 5A). Similarly, enhanced root bending has been shown following LatB treatment in *Arabidopsis* roots [42]. Depolymerization of the actin cytoskeleton inhibits PIN2 trafficking to the vacuole [8]; however, trafficking to the vacuole remains functional in the presence of TE1 (Figures 3C and 3D). Thus it is unlikely that the cellular effects caused by TE1 are due to its direct effect on the actin cytoskeleton.

To further distinguish between the effects of TE1 and LatB, two *Arabidopsis* accessions, Sha and HKT2-4, were exploited. These accessions were selected as being partially resistant to multiple effects of TE1 including agravitropic growth, inhibition of endocytosis, formation of TE1 bodies and growth inhibition (Figure 5, and Supplementary Figures S9 and S10), suggesting a common mechanism underlying these effects. However, both Sha and HKT2-4 were similarly sensitive to the effect of 50 nM LatB on root growth and all four ecotypes tested showed enhanced root bending in the presence of LatB (Figure 5).

In summary, we show that compound TE1 inhibits endocytosis and causes a rapid accumulation of PIN2–GFP and other membrane proteins within a BFA-sensitive PVC-related compartment. The increased appearance of GFP within the vacuole following longer treatment is consistent with disruption of retrieval from the PVC to TGN-mediated trafficking. Thus the mode of action is distinct from other known small molecule modulators of endomembrane trafficking (for reviews, see [1,10]). This small molecule can be a valuable additional tool to dissect complex trafficking mechanisms within the endomembrane system, endocytosis and the TGN recruitment pathway in particular, in plants and perhaps in other systems. The availability of naturally resistant *Arabidopsis* accessions also provides a potential genetic route to identifying the molecular target.

## Online data

Supplementary data
