# Supplementary material for: Trafficking modulator TENin1 inhibits endocytosis, causes endomembrane protein accumulation at the pre-vacuolar compartment and impairs gravitropic response in Arabidopsis thaliana
Source: Biochem J. 2014 May 13;460(Pt 2):177–85. doi: 10.1042/BJ20131136 (PMC4100570; doi:10.1042/BJ20131136)
Supplement: Supplementary data [file bj4600177add.pdf]

Biochem. J. (2014) **460**, 177–185 (Printed in Great Britain) doi:10.1042/BJ20131136

online data

SUPPLEMENTARY ONLINE DATA

Trafficking modulator TENin1 inhibits endocytosis, causes endomembrane protein accumulation at the pre-vacuolar compartment and impairs gravitropic response in *Arabidopsis thaliana*

Rupesh PAUDYAL*, Adam JAMALUDDIN*, James P. WARREN†, Siamsa M. DOYLE‡, Stéphanie ROBERT‡, Stuart L. WARRINER† and Alison BAKER*[1]

*Centre for Plant Sciences, University of Leeds, Leeds LS2 9JT, U.K.
†School of Chemistry, Faculty of Mathematics and Physical Sciences, University of Leeds, Leeds LS2 9JT, U.K.
‡Umeå Plant Science Centre, Department of Forest Genetics and Plant Physiology, Swedish University of Agricultural Sciences (SLU), Umeå 90183, Sweden

EXPERIMENTAL

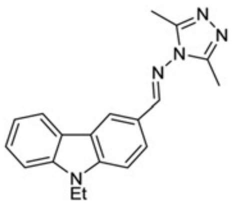

Chemical synthesis of TENin1

4-Amino-3,5-dimethyl-1,2,4 triazole (47.5 mg, 0.45 mmol, 1 eq) was dissolved in dry ethanol (0.36 ml). 3-Formyl-*N*-ethyl-carbazole (100 mg, 0.45 mmol, 1 eq) was dissolved in dry ethanol (0.36 ml) and then added dropwise, after which a catalytic amount of concentrated HCl was added, the solution was stirred for 60 min under N_2 and then poured on to ice. The precipitate was extracted with three 50 ml volumes of DCM (dichloromethane) and the organic layers were collected and washed three times with 20 ml of brine, dried ($MgSO_4$) and then concentrated to leave a crude product which was purified by flash chromatography using 9:1 DCM/methanol as the eluent to give TENin1 as off-white spindles. Yield, 12 mg; R_F, 0.67 (9:1 DCM/methanol). ^{1}H NMR (300 MHz, [^{2}H]chloroform) δ_H 8.56 (2 H, s, ArH, where ArH indicates an aromatic hydrocarbon, hydrazone-H), 8.17 (1 H, d, *J* 7.7 Hz, ArH), 8.02 (1 H, dd, *J* 8.7, 1.4 Hz, ArH), 7.56–7.47 (3 H, m, ArH), 7.34 (1 H, td, *J* 7.9, 0.9 Hz ArH), 4.44 (2 H, q, *J* 7.2 Hz, CH_2–CH_3), 2.52 (6 H, s, CH_3, triazole), 1.49 (3 H, t, *J* 7.2 Hz, CH_2–CH_3), ^{13}C NMR (75 MHz, [^{2}H]choloroform), δ_C 165.7, 148.1, 142.8, 140.6, 126.9, 126.2, 123.5, 122.8, 122.7, 122.4, 120.8, 120.3, 109.2, 38.0 (CH_2–CH_3), 13.9 (CH_2–CH_3), 11.28 (CH_3, triazole). V_{max}/cm 3057 (C–H) 1626 (C = N) 1461 (C = C aromatic). ESI + found M^+H^+ 318.1709 $C_{19}H_{20}N_5{}^+$ requires 318.1713.

Synthesis of analogues

TENin1 analogues (see Figure S5) were prepared using similar methods to that listed above. *N*-allyl analogues were prepared by allylation of carbazole under basic conditions [1] and Vilsmeier formylation [2] followed by hydrazone formation.

Immunolocalization

Endomembrane marker lines expressing NAG–GFP, VHaA1–GFP and GFP–ARA7 were treated with DMSO or 25 μM TE1 and fixed in 4 % formaldehyde and were washed three times each with Triton X-100/PBS and Triton X-100/water followed by incubation at 37 °C in driselase for 30 min. They were then washed three times with Triton X-100/PBS for 15 min each, twice with Igepal for 30 min and three times with Triton X-100/PBS 15 min, followed by blocking with 3 % BSA for 60 min. Samples were incubated with an anti-PIN2 antibody (1:1000 dilution) raised in rabbits at 37 °C for 4 h and washed five times in three times with Triton X-100/PBS for 15 min. Following a 4-h incubation with an anti-rabbit–CY3 (indocarbocyanine) antibody (1:250 dilution) raised in goats (Stratech) at 37 °C, lines were washed three times with Triton X-100/PBS for 15 min and finally three times with water for 15 min. The samples were then ready to be visualized under confocal microscope.

[1] To whom correspondence should be addressed (email a.baker@leeds.ac.uk).

© 2014 The Author(s)
This is an Open Access article distributed under the terms of the Creative Commons Attribution Licence (CC-BY) (http://creativecommons.org/licenses/by/3.0/) which permits unrestricted use, distribution and reproduction in any medium, provided the original work is properly cited.

Biochemical Journal www.biochemj.org

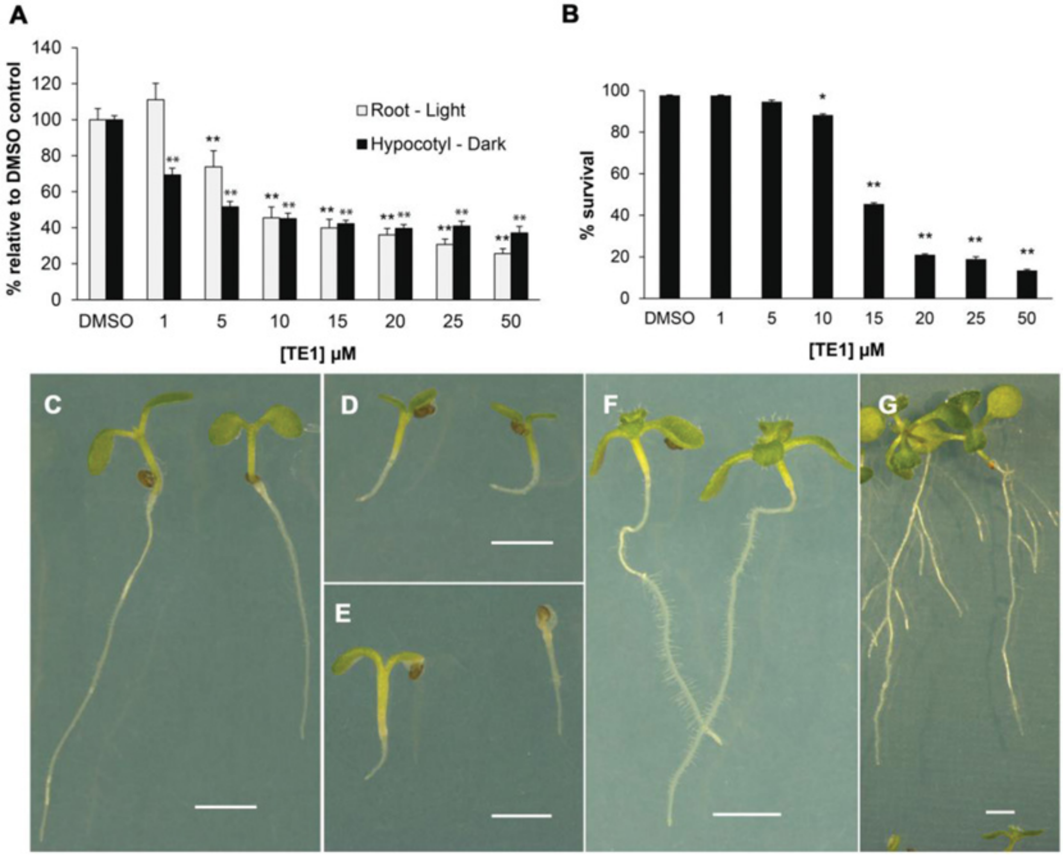

Figure S1 Effect of TE1 on root growth and survival of *Arabidopsis* seedlings

(**A** and **B**) *Arabidopsis* seedlings were grown for 7 days. (**A**) Root lengths of seedlings grown in the light (grey bars) and hypocotyl lengths of seedlings grown in the dark (black bars) are shown. Plant growth grown on TE1 was standardized against a DMSO control. Data are merged from three repetitions. For root growth 60–143 seedlings and for hypocotyl growth 80–195 seedlings per condition were measured. Error bars represent the S.E.M. $*P \leqslant 0.05$ and $**P \leqslant 0.01$. (**B**) Percentage survival of seedlings in the presence of TE1. Data are merged from three repetitions and a total of 75–195 seedlings were scored per condition. Error bars represent the S.E.M. $*P \leqslant 0.05$ and $**P \leqslant 0.01$. Seedlings (5-day-old) grown in medium containing DMSO (**C**) and 25 μM TE1 (**D**). Plants transplanted from DMSO after 5 days to fresh DMSO-containing medium for further 5 days (**G**), or from TE1 to TE1 for further 5 days (**E**) or recovered from TE1 to DMSO (**F**). Scale bars, 2 mm.

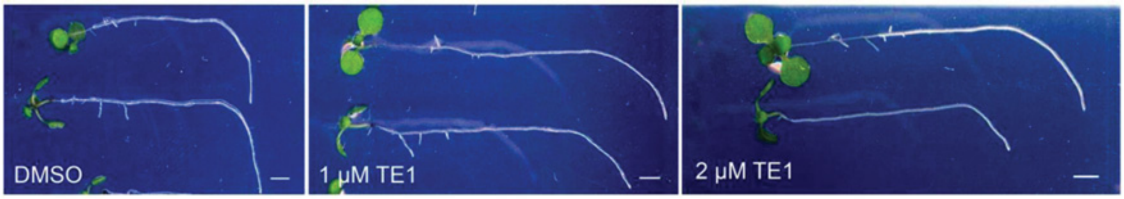

Figure S2 TE1 inhibits root gravitropic response

Seedlings (6-day-old) grown in DMSO-containing medium were transplanted to medium containing DMSO, 1 μM TE1 or 2 μM TE1 and gravistimulated for 48 h. Scale bars, 5 mm.

© 2014 The Author(s)
This is an Open Access article distributed under the terms of the Creative Commons Attribution Licence (CC-BY) (http://creativecommons.org/licenses/by/3.0/) which permits unrestricted use, distribution and reproduction in any medium, provided the original work is properly cited.

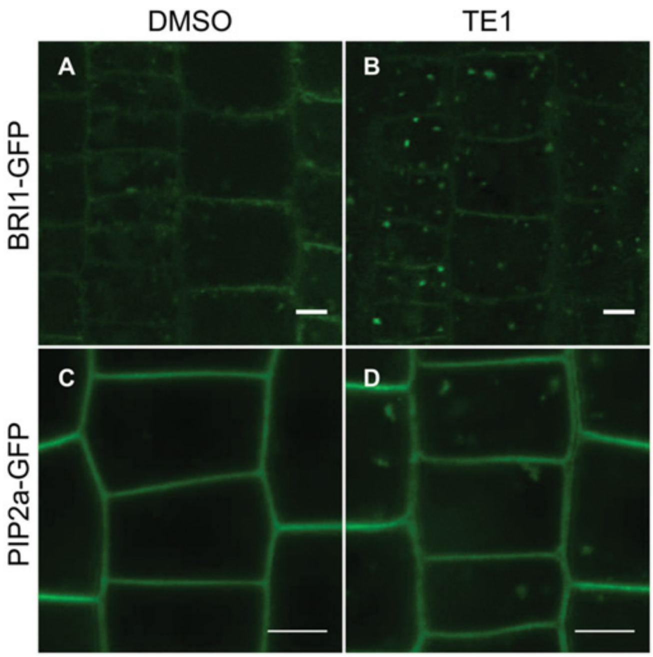

Figure S3 TE1 also interrupts trafficking of other membrane proteins

Seedlings expressing BRI1–GFP incubated for 180 min in DMSO (**A**) and 25 μM TE1 (**B**) and PIP2a–GFP incubated for 120 min in DMSO (**C**) and 25 μM TE1 (**D**). Scale bars, 5 μm.

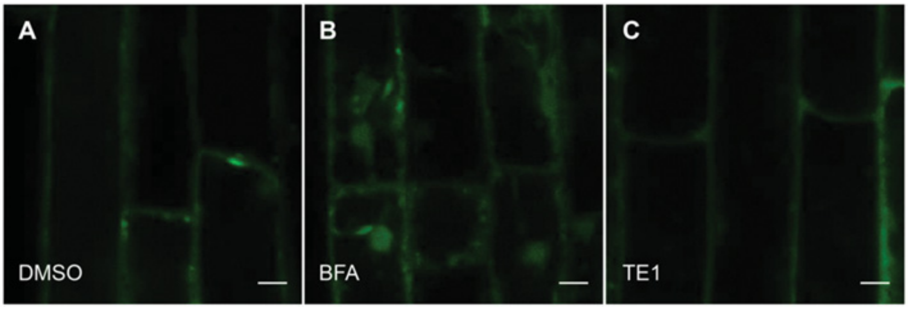

Figure S4 Secretion of newly synthesized protein is functional in the presence of TE1

Seedlings (7-day-old) expressing secGFP protein incubated in DMSO (**A**), 50 μM BFA (**B**) and 25 μM TE1 (**C**) for 120 min. Scale bars, 5 μm.

© 2014 The Author(s)
This is an Open Access article distributed under the terms of the Creative Commons Attribution Licence (CC-BY) (http://creativecommons.org/licenses/by/3.0/) which permits unrestricted use, distribution and reproduction in any medium, provided the original work is properly cited.

Chemical	Structure	PIN2-GFP after 120 min at 25 μM chemical	Active
DMSO		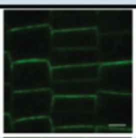	
TE1<br>$C_{19}H_{19}N_5$<br>(317.4)	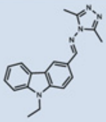	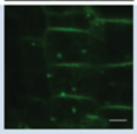	Yes
JW4<br>$C_{14}H_{13}N$<br>(195.26)	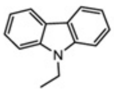	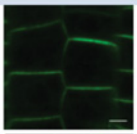	No
JW30<br>$C_{20}H_{19}N_5$<br>(329.4)	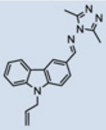	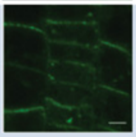	Yes
JW32<br>$C_{17}H_{15}N_5$<br>(289.3)	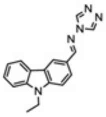	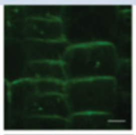	Yes
JW35<br>$C_{19}H_{21}N_3$<br>(291.39)	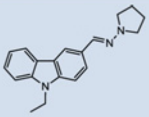	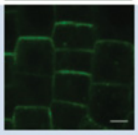	Yes
JW42<br>$C_{19}H_{21}N_3O$<br>(307.39)	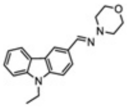	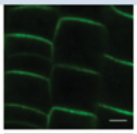	No
JW45<br>$C_{10}H_9NO$<br>(159.18)	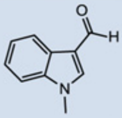	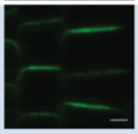	No
JW47<br>$C_{15}H_{17}N_5$<br>(267.33)	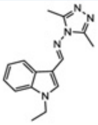	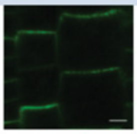	No
JW48<br>$C_{21}H_{21}N_5O$<br>(359.42)	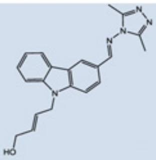	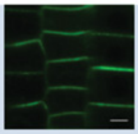	No

© 2014 The Author(s)
This is an Open Access article distributed under the terms of the Creative Commons Attribution Licence (CC-BY) (http://creativecommons.org/licenses/by/3.0/) which permits unrestricted use, distribution and reproduction in any medium, provided the original work is properly cited.

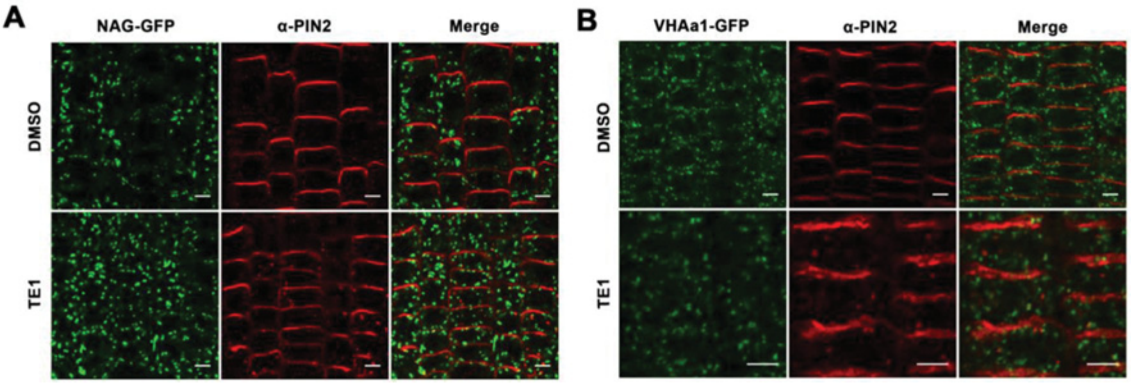

Figure S6 Immunolocalization studies using an anti-PIN2 antibody

Seedlings (7-day-old) expressing NAG–GFP (**A**) and VHAa1–GFP (**B**) were incubated in DMSO (upper row) and 25 μM TE1 (lower row) for 120 min and promptly fixed in 4 % formaldehyde. Anti-PIN2 antibody was used for immunodetection of PIN2 proteins (middle columns). Merged images of anti-PIN2 antibody labelling and respective endomembrane markers can be seen in the third column of each panel. Scale bars, 5 μm.

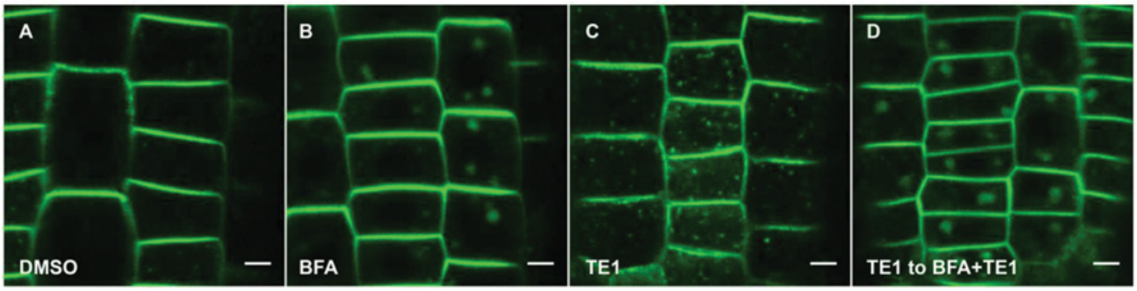

Figure S7 TE1 bodies are BFA-sensitive

Seedlings expressing PIN2–GFP were incubated in DMSO for 120 min (**A**), 50 μM BFA for 60 min (**B**), 25 μM TE1 for 120 min (**C**) or 50 μM BFA plus 25 μM TE1 for 60 min following a 60-min pre-incubation at 25 μM TE1 (**D**). Scale bars, 5 μm.

Figure S5 SAR study of TE1 analogues

SAR study to identify functional group required biological activity of TE1 using PIN2–GFP. Seedlings were incubated in 25 μM chemical for 120 min and intracellular accumulation of PIN2–GFP was monitored to determine the bioactivity. Scale bars, 5 μm.

© 2014 The Author(s)
This is an Open Access article distributed under the terms of the Creative Commons Attribution Licence (CC-BY) (http://creativecommons.org/licenses/by/3.0/) which permits unrestricted use, distribution and reproduction in any medium, provided the original work is properly cited.

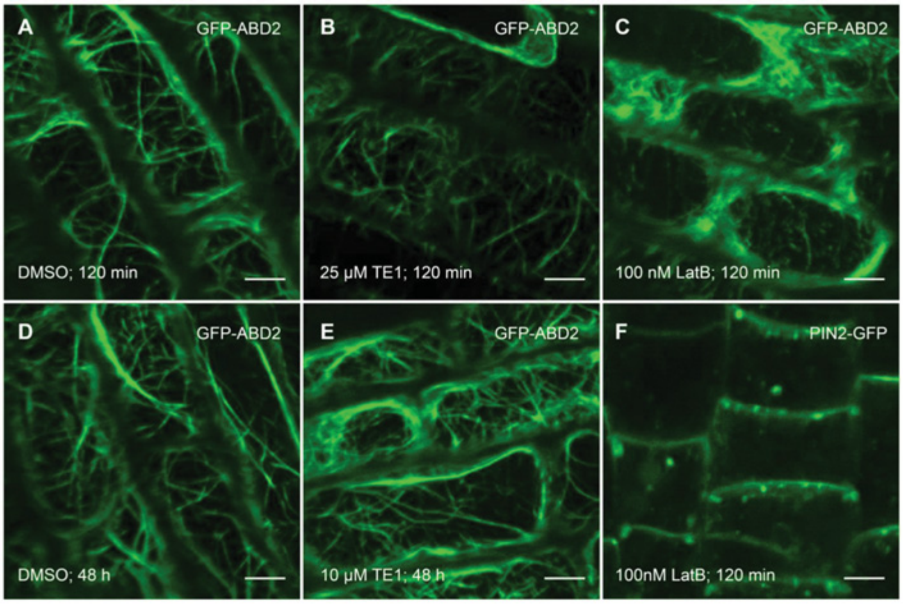

Figure S8 Prolonged exposure to a low concentration of TE1 does not visibly affect the actin cytoskeleton

Arabidopsis seedlings expressing GFP–ABD2 (**A**–**E**) were incubated in DMSO-containing medium for 120 min (**A**) and 48 h (**D**). GFP–ABD2 seedlings were also incubated in 25 μM TE1 for 120 min (**B**), 10 μM TE1 for 48 h (**E**) or 100 nM LatB for 120 min (**C**). Seedlings expressing PIN2–GFP incubated in 100 nM LatB for 120 min (**F**) is also shown. Scale bars, 5 μm.

© 2014 The Author(s)
This is an Open Access article distributed under the terms of the Creative Commons Attribution Licence (CC-BY) (http://creativecommons.org/licenses/by/3.0/) which permits unrestricted use, distribution and reproduction in any medium, provided the original work is properly cited.

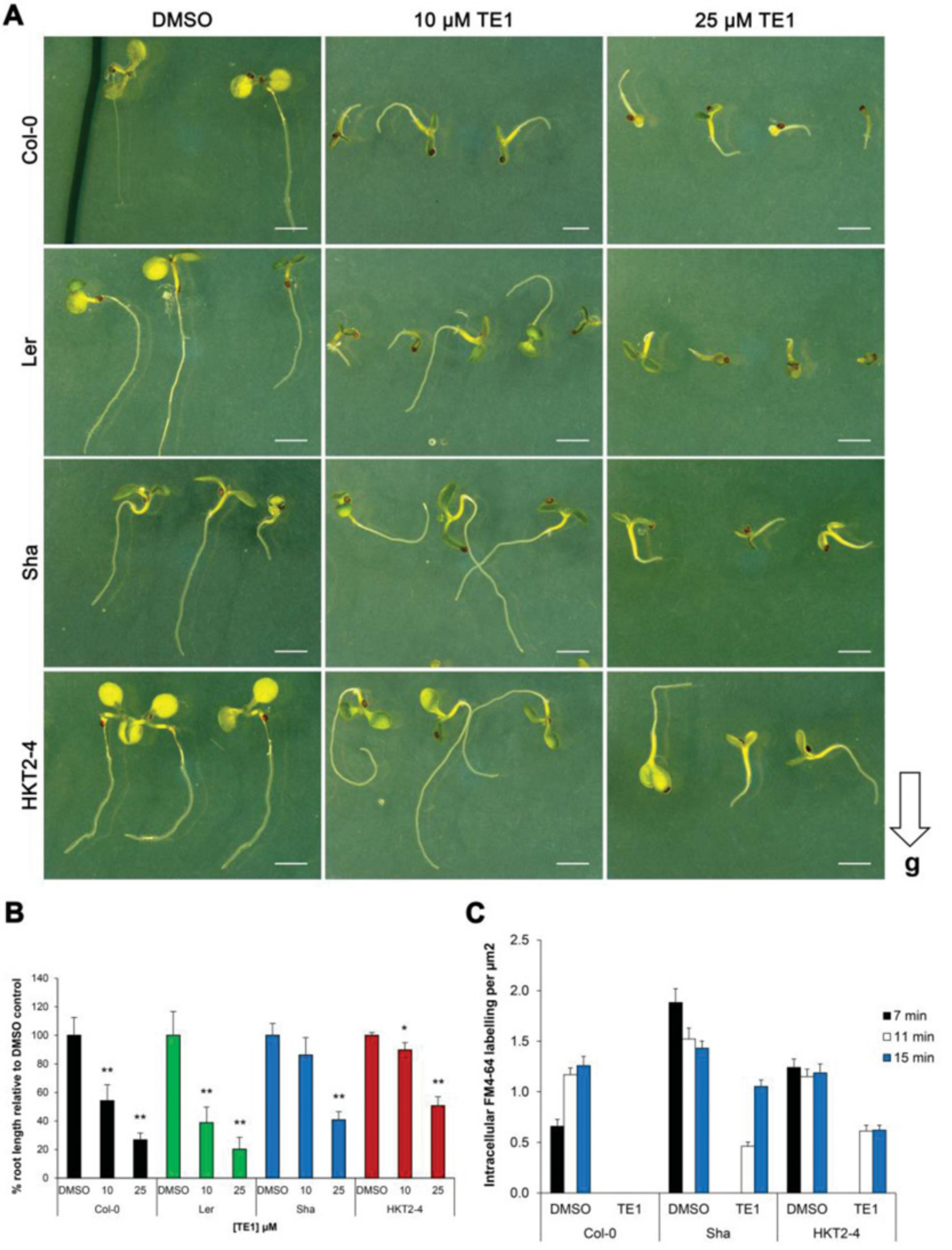

Figure S9 ***Arabidopsis*** **natural accessions display differential sensitivity to TE1**

(**A**) *Arabidopsis* (7-day-old) accessions, Col-0 (first row), L*er* (second row), Sha (third row) and HKT2-4 (fourth row), grown in medium containing DMSO (left-hand column), 10 μM TE1 (middle column) or 25 μM TE1 (right-hand column). Arrow indicates direction of gravity vector. Scale bars, 2 mm. (**B**) Root growth of 7-day-old *Arabidopsis* accessions grown in DMSO, 10 μM TE1 or 25 μM TE1. Root lengths were standardized against the DMSO control. Error bars represent the S.E.M. $*P \leqslant 0.05$ and $**P \leqslant 0.01$. (**C**) Quantification of number of intracellular labelling of FM4-64 per μm^2 in seedlings treated with DMSO or 25 μM TE1 for 120 min. FM4-64 uptake was quantified 7, 11 and 15 min after FM4-64 incubation.

© 2014 The Author(s)
This is an Open Access article distributed under the terms of the Creative Commons Attribution Licence (CC-BY) (http://creativecommons.org/licenses/by/3.0/) which permits unrestricted use, distribution and reproduction in any medium, provided the original work is properly cited.

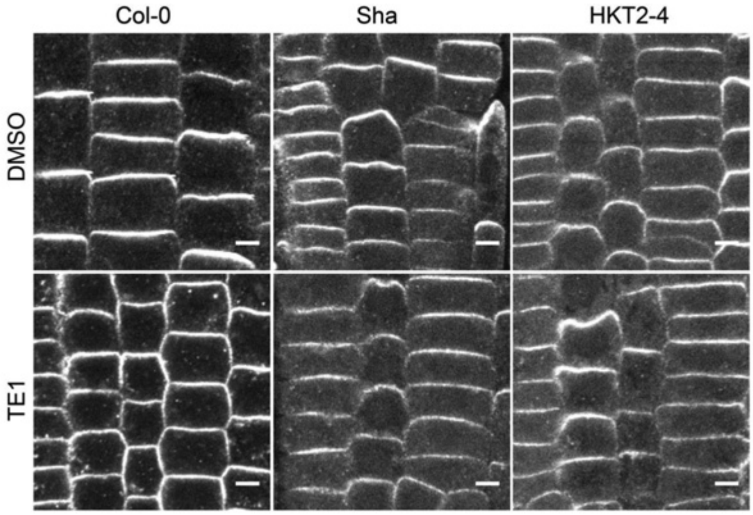

Figure S10 ***A. thaliana*** **Sha and HKT2-4 plants are more resistant to the induction of TE1 bodies**

Seedlings (7-day-old) were respectively incubated in DMSO-containing medium (upper row) or 25 μM TE1 (lower row) for 120 min and promptly fixed in 4 % formaldehyde. Anti-PIN2 antibody was used for immunodetection of PIN2 proteins in *A. thaliana* ecotypes, Col-0 (left-hand column), Sha (middle column) and HKT2-4 (right-hand column). Scale bars, 5 μm.

© 2014 The Author(s)
This is an Open Access article distributed under the terms of the Creative Commons Attribution Licence (CC-BY) (http://creativecommons.org/licenses/by/3.0/) which permits unrestricted use, distribution and reproduction in any medium, provided the original work is properly cited.

Table S1 List of the *Arabidopsis* natural accessions screened against TE1

The Nottingham Arabidopsis Stock Centre (NASC) code and the accession name are shown in the first column and the second column respectively. Third, fourth and fifth columns show the primary screen for growth/germination, gravitropism and re-screening to confirm gravitropism respectively. √, passed therefore taken forward to next screen; ×, failed therefore dropped from the screen; *, used as control even if it did not pass through the screen.

NASC code	Name	Primary	Secondary	Tertiary
N1092	Col-0	√*	×*	×*
N1642	L*er*	√*	×*	×*
N1601	Ws2	√	×	
N76347	Aitba-2	×		
N76348	Toufl-1	×		
N76349	Vezzano-2	×		
N76350	Vezzano-2	×		
N76351	Rovero-1	×		
N76352	Voeran-1	×		
N76353	Altenb-2	√	√	×
N76354	Mitterberg-1	√	√	×
N76355	Castelfed-4	√	√	×
N76356	Castelfed-4	√	√	×
N76357	Bozen-1	√	√	×
N76358	Bozen-1	√	√	×
N76359	Ciste-1	×		
N76360	Ciste-2	×		
N76361	Monte-1	×		
N76362	Angel-1	×		
N76363	Moran-1	√	×	
N76364	Mammo-2	√	×	
N76365	Mammo-1	×		
N76366	Angit-1	×		
N76367	Lago-1	×		
N76368	Apost-1	×		
N76369	Dobra-1	×		
N76370	Petro-1	√	√	×
N76371	Lecho-1	√	√	×
N76372	Jablo-1	×		
N76373	Bolin-1	√	√	×
N76374	Shigu-2	√	√	×
N76375	Shigu-1	×		
N76376	Kidr-1	√	×	
N76377	Stepn-2	×		
N76378	Stepn-1	√	×	
N76379	Sij1	√	×	
N76380	Sij2	√	×	
N76381	Sij4	×		
N76382	Sha	√	√	√
N76383	Koz2	√	×	
N76384	Kly4	×		
N76385	Kly1	√	×	
N76386	Dog-4	√	×	
N76387	Xan-1	×		
N76388	Lerik1-3	×		
N76389	Istisu-1	√	×	
N76390	Lag2-2	√	×	
N76391	Vash-1	×		
N76392	Bak-2	√	√	×
N76393	Bak-7	×		
N76394	Yeg-1	√	×	
N76395	Kastel-1	×		
N76396	Koch-1	√	×	
N76397	Del-10	×		
N76398	Nemrut-1	×		
N76399	Ey1.5-2	√	√	×
N76400	Star-8	×		
N76401	Tu-Scha-9	×		
N76402	Nie1-2	√	√	×
N76403	Tu-SB30-3	√	×	
N76404	HKT2-4	√	√	√
N76405	Tu-Wa1-2	×		
N76406	Ru3.1-31	√	√	×
N76407	Tu-V-13	×		
N76408	Wal-HasB-4	×		
N76409	Agu-1	×		
N76410	Cdm-0	×		
N76411	Don-0	×		
N76412	Fei-0	√	×	
N76413	Leo-1	×		
N76414	Mer-6	×		
N76415	Ped-0	×		
N76416	Pra-6	×		
N76417	Qui-0	×		
N76418	Vie-0	×		
N76419	Slavi-1	√	√	×
N76420	Copac-1	×		
N76421	Borsk-2	√	√	×
N76422	Krazo-2	√	√	×
N76423	Galdo-1	×		
N76424	Timpo-1	×		
N76425	Valsi-1	√	√	×
N76426	Leb-3	√	×	

REFERENCES

1 Daeffler, C. S. and Grubbs, R. H. (2011) Radical-mediated anti-Markovnikov hydrophosphonation of olefins. Org. Lett. **13**, 6429–6431 CrossRef PubMed

2 Bian, L., Sun, G., Sun, Y. and Tang, W. (2012) Synthesis of sterically hindered antipyrines for intramolecular charge transfer facilitated sensing. Phys. Org. Chem. **25**, 1112–1118 CrossRef

Received 27 August 2013/25 February 2014; accepted 21 March 2014
Published as BJ Immediate Publication 21 March 2014, doi:10.1042/BJ20131136

© 2014 The Author(s)
This is an Open Access article distributed under the terms of the Creative Commons Attribution Licence (CC-BY) (http://creativecommons.org/licenses/by/3.0/) which permits unrestricted use, distribution and reproduction in any medium, provided the original work is properly cited.
